# Protective Effect of Prim-O-Glucosylcimifugin on Ulcerative Colitis and Its Mechanism

**DOI:** 10.3389/fphar.2022.882924

**Published:** 2022-05-18

**Authors:** Yu Yin, Kunjian Liu, Guofeng Li

**Affiliations:** College of Traditional Chinese Medicine, Changchun University of Chinese Medicine, Changchun, China

**Keywords:** POG, ulcerative colitis, AKT, NF-κB, MAPK

## Abstract

Intestinal epithelial immune dysfunction or imbalance in the homeostasis of intestinal flora can lead to the occurrence or exacerbation of ulcerative colitis (UC). Prim-O-glucosylcimifugin (POG) is an extract of Chinese traditional medicine (TCM) Saposhnikov, which has analgesic, anti-inflammatory, and antioxidant effects. The present work discussed how the POG alternated ulcerative colitis (UC) along with its underlying mechanism. This was clarified by performing animal studies in a mice model, wherein UC was induced by dextran sulfate sodium (DSS). *In vivo* studies have found that POG increased clinical score, colonic length, and weight of mice in the ulcerative colitis model. It repaired the pathological injury of an intestinal mucosa within mice while inhibiting the inflammatory factor levels such as IL-1β, TNF-α, and IL-6. Meanwhile, by16SrDNA sequencing analysis, it was found that POG regulated the richness of intestinal microbiota structure and repaired the intestinal immune barrier by upregulating the expression levels of tight junction proteins Occludin, Claudin-3, and ZO-1. To further confirm the above results, we found in *in vitro* studies that POG also protected lipopolysaccharide- (LPS-) induced RAW264.7 cells. POG dramatically suppressed inflammatory factor production (including TNF-α, IL-1β, and IL-6) within LPS-treated RAW264.7 cells by inhibiting the activation of ERK1/2, AKT, JNK1/2, IκB-α, P38, and P65 phosphorylation. In conclusion, POG plays a protective role against UC by inhibiting the activation of pro-inflammatory signaling pathways MAPK, AKT, and NF-κB; repairing the integrity of the intestinal barrier; and regulating the diversity and abundance of intestinal flora.

## Introduction

Ulcerative colitis (UC) represents the non-specific, chronic inflammatory bowel disease (IBD) whose cause remains unclear. It is difficult to control due to its low cure rate and high recurrence rate. UC has the typical manifestations of rectal urgency, bloody diarrhea with/without mucus, abdominal pain to varying degrees (usually improved after defecation), and tenesmus (Leikin, 2019). Recently, several studies have confirmed that the intestinal immune barrier and intestinal flora in the host intestinal mucosa and ulcerative colitis are closely related. The onset of UC will inevitably damage the intestinal barrier (Pan et al., 2020). Serious injuries to a mucosal structure or even a little alteration of barrier component may comprise functions of the intestinal barrier (Nalle and Turner, 2015). These changes in intestinal barrier function can promote the movement of the intestine, force bacteria to not stay in local intestinal mucosa for a long time, and start the self-cleaning function of the intestinal cavity (Camilleri, 2019; He et al., 2019). Therefore, it is essential to maintain the integrity of the intestinal barrier and the homeostasis of the intestinal flora for the treatment of UC (Quigley, 2016).

Aminosalicylates, corticosteroids, and immunosuppressants are common UC treatments, but only half of the patients achieve remission (Chen et al., 2018). The gut microbiota plays an important role in maintaining enteric homeostasis (Zhou et al., 2020). Beneficial bacteria form a natural barrier to the entire intestine, whereas harmful bacteria disrupt the intestinal environment, damage the intestinal epithelial cells, and destroy the intestinal structure (Kelly et al., 2015). The colonization and imbalance of harmful flora have been shown in studies to directly damage the intestinal epithelial barrier and promote the aggravation of UC (Pickard et al., 2017) symptoms. More drugs to protect the intestinal immune barrier and maintain intestinal homeostasis are urgently needed.

At present, the application of Chinese medicine extract to ulcerative colitis has an obvious intervention effect, and more and more people recognize the importance of its therapeutic effects in Prim-O-glucosylcimifugin (POG), which is an extract of traditional Chinese medicine Saposhnikova (Wu et al., 2016). POG has been suggested to have anti-inflammation, analgesia, antioxidation, and anticancer activities. They are used in the treatment of colds, headaches, rheumatoid arthritis (RA), myeloma, and breast cancer (Wu et al., 2016; Zhou et al., 2017; Gao et al., 2019). Based on anti-inflammatory functions, this study explores the role of POG in repairing the intestinal immune barrier and regulating intestinal flora to maintain intestinal environmental homeostasis (Xu et al., 2018), to better clarify the authenticity and effectiveness of POG.

## Materials and Methods

### Reagents

This work acquired POG (B21157–20 mg) (purity>98%) in Shanghai Yuanye Bio-Technology Co., Ltd. (Shanghai, China). DMEM (SH30285. FS), 0.05% trypsin (SH30236.01), PBS solution (SH30256.01), fetal bovine serum (SV30087.03), penicillin, and streptomycin (SV30087.03) were purchased from HyClone Laboratories (UT, United States). We acquired TRIzol (93289), DMSO (D2650), LPS (L-2880), Evans Blue (E2129), and formamide (F7503) from Sigma-Aldrich (Saint Louis, MO, United States). Primary antibodies, including ERK1/2 (9102), p-ERK1/2 (9101), p-AKT (4060), AKT (4691), p38 (9212), p-P38 (9211), NF-κB p65 (4764), NF-κB p-p65 (3033), NF-κB IκB-α (L35A5), NF-κB p-IκB-α (ser32) JNK (9252), p-JNK (4668), β-actin (3700), COX-2 (ab62331), iNOS (ab178945), ZO-1 (D6L1E), Occludin (ab216327), and Claudin-3 (ab15102) were provided by Abcam (Cambridge, United Kingdom) or Cell Signaling Technology (MA, United States). In addition, the goat anti-mouse/rabbit secondary antibody was obtained from Boster Biological Technology (CA, United States). This work acquired ELISA kits for TNF-α (430907), IL-6 (431307), and IL-1β (432604) from Biolegend (San Diego, CA92121, United States). Reverse transcription kits (OligdT, RRI, dNTP Mix 10 mM, MLV, MLV Buffer) and 2 × SYBR Premix were provided by Takara Biomedical Technology Co., Ltd. (Kyoto, Japan).

### Drug Management

In order to carry out the experiment, POG was dissolved in purified water and stored. The safe dosage was screened before the experiment, and it was found that 2.5, 5, and 10 mg/kg were safe for mice. Therefore, we chose 2.5/5/10 mg/kg of POG for animal experiments, and 12.5, 25, and 50 μmol/ml of POG were selected for the cell line studies.

### Animal Experiments

Each animal experiment was carried out according to the legal regulations. The study protocols were approved by the Institutional Animal Management and Use Committee of Jilin University (Changchun, China) as per the protocol (Permit Number: SY202201008). This work obtained 6–8-week-old male C57BL/6 mice applied from Liaoning Changsheng Biotechnology Co., Ltd. In the experiment, 36 mice were randomized and grouped into six groups. Animals were divided into DSS, POG, control, DSS + POG (2.5, 5, and 10 mg/kg) groups, with six each. Three days before the experiment, all animals had free access to water and food. POG and DSS + POG groups (2.5, 5, and 10 mg/kg) were given corresponding doses of POG by intraperitoneal injection. After 3 days, all the groups were free to eat, the control group and POG group were free to drink, and the other groups were given 2% DSS (2 g added with 100 ml purified water). The POG and DSS + POG groups (2.5, 5, and 10 mg/kg) were given the corresponding dose of POG by intraperitoneal injection. Mice were euthanized on the 10th day after successful modeling for seven consecutive days. The specific experiments are as follows.

### Cell Culture and Activity Determination

This work cultivated RAW264.7 cells within DMEM that contained 10% fetal bovine serum (FBS) under 37°C and 5% carbon dioxide (CO_2_) conditions. The suitable concentration of the cell drug was selected by CCK8 assay. After counting, cells were inoculated into 96-well plates with 100 µL per well at the logarithmic growth stage. LPS stimulation was added for 2 h after pre-administration for 1 h, and drug treatment for 24 h, then the supernatant was discarded, and all 110 µL CCK8 dilution (Beyotime Inst Biotech, Beijing, China) wells were added to the wells. Following washing, OD _(450nm)_ values were detected after 3 h.

### qRT-PCR Assay

LPS and drugs were used to treat RAW264.7 cells to extract their mRNA, and cellular IL-1β, TNF-α, and IL-6 levels were analyzed by the qRT-PCR method according to the prior description (Hao et al., 2020). Table 1 displays all substrates utilized.

**TABLE 1:** Primers utilized in qRT-PCR.

Gene	Sequence	Length (bp)
β-Actin	F: 5′-GTC​AGG​TCA​TCA​CTA​TCG​GCA​AT-3′	147
	R: 5′-AGA​GGT​CTT​TAC​GGA​TGT​CAA​CGT-3′	
IL-1β	F: 5′-TGT​GAT​GTT​CCC​ATT​AGA​C-3′	139
	R: 5′-AAT​ACC​ACT​TGT​TGG​CTT​A-3′	
IL-6	F: 5′-AGC​CAC​TGC​CTT​CCC​TAC-3′	138
	R: 5′-TTG​CCA​TTG​CAC​AAC​TCT​T-3′	
TNF-α	F: 5′-CCA​CGC​TCT​TCT​GTC​TAC​TG-3′	136
	R: 5′-CCA​CGC​TCT​TCT​GTC​TAC​TG-3′	

### Disease Activity Index

The body weight (BW), fecal occult blood and stool character, and DAI were determined using a scoring system (Table 2) according to the previous description (Tian et al., 2016).

**TABLE 2:** Clinical scoring system.

Score	Bodyweight decrease rate	Fecal property	Hematochezia status
0	0%	Normal	Normal
1	1%–5%	Semi-loose (+)	Feces with occult blood (+)
2	6%–10%	Semi-loose (++)	Feces with occult blood (++)
3	1%–15%	Loose (+)	Bloody feces (+)
4	>15%	Loose (++)	Bloody feces (++)

### ELISA

The expression levels of inflammatory factors TNF-α, IL-1β, and IL-6 in the colon tissues of mice were measured as per the instructions given on the Elisa kit.

### Clinical Scoring and Sample Collection

The mice were weighed and scored clinically according to the scoring criteria in Table 2. Finally, the colon and feces of the mice were collected for relevant experimental analysis.

### Hematoxylin and Eosin Staining

After collecting mice colonic samples, they were fixed with 4% formaldehyde, paraffin-embedded tissue, followed by slicing into 5 µm sections and H&E staining. Histology was observed by an optical microscope after staining. Finally, histological scores were performed according to Table 3. The specific methods were referred to from the reported method (Zhang et al., 2021).

**TABLE 3:** Histological scoring criteria.

Score	Mucosal architecture	Cellular infiltration	Goblet cell depletion
0	Absent	None	Absent
1	Mild	Infiltrate around the crypt basis	Present
2	Medium	Infiltrate reaching the muscularis mucosae	
3	Severe	Infiltrate reaching the submucosa	

### Immunofluorescence Staining

About 5 µm tissue sections were dewaxed and dehydrated with ethanol gradient, antigen retrieval, and rinsed by PBS for 5 min thrice. 5% donkey serum resulted in blocked sections for 30 min. The primary antibody [ZO-1 (1:100), Occludin (1:100), and Claudin-3 (1:100)] was supplemented dropwise and kept at 4°C in the refrigerator overnight. It was washed with PBS 3 times (5 min each time). After 1 h at room temperature, binding to goat anti-rabbit IgG antibody (1:2000, Santa Cruz), cells were washed thrice and stained with DAPI.

### Western Blotting Assay

This work extracted total colonic tissue protein using RIPA lysate after grinding. The protein concentration of mice colon tissues was determined using a BCA reagent (Beibo Biological Technology, shanghai, China). The proteins (15 µg) were later separated by 12% SDS-PAGE, followed by transfer onto PVDF membranes (Millipore, Darmstadt, Germany). Thereafter, 5% defatted milk was utilized to block PVDF membranes at room temperature for a 2 h period. PVDF membrane and primary antibody ERK1/2 (1:1000), p-ERK1/2 (1:1000), p-AKT (1:1000), AKT (1:1000), p38 (1:1000), p-P38 (1:1000), NF-κB p65 (1:1000), NF-κB p-p65 (1:1000), NF-κB IκB-α (1:1000), NF-κB p-IκB-α (1:1000) JNK (1:1000), p-JNK (1:1000), β-actin (1:1000), COX-2 (1:1000), iNOS (1:1000), ZO-1 (1:1000), Occludin (1:1000), and Claudin-3 (1:1000) were subject to overnight preservation under 4°C and rinsing by tbst lotion (Guo et al., 2019). Then, the PVDF membrane was subject to 2 h incubation with goat anti-mouse/rabbit secondary antibody (1:5,000) and washed in the TBS-T washing solution. Then, enhanced chemiluminescence solution was used to detect the changing trend of protein bands.

### 16S rDNA High-Throughput Sequencing

The Illumina Novaseq sequencing platform is used to examine microbial diversity. A small fragment library was created using paired-end sequencing to carry out HTS. In order to compare sample species composition, abundance and annotation analyses of species were performed, as well as filtering, clustering, or denoising of reads splicing. α-Diversity, β-diversity, correlation analysis, and significant species difference analysis were used to examine inter-sample differences. Beijing Biomark Biology Co., Ltd. completed the bacterial population sequencing analysis.

### Statistical Analysis

The results were presented as mean ± standard deviation (SD) deviation. Unpaired Student’s *t*-tests were used in Prism 8.0 to compare two groups, while general linear model ANOVA was used in Prism 8.0.20 to compare multiple groups. Pathological and clinical scores were evaluated using non-parametric tests (Liu K. et al., 2020).

## Results

### POG Has a Good Preventive and Protective Effect on the DSS-Induced UC Mice Model

**FIGURE 1:**
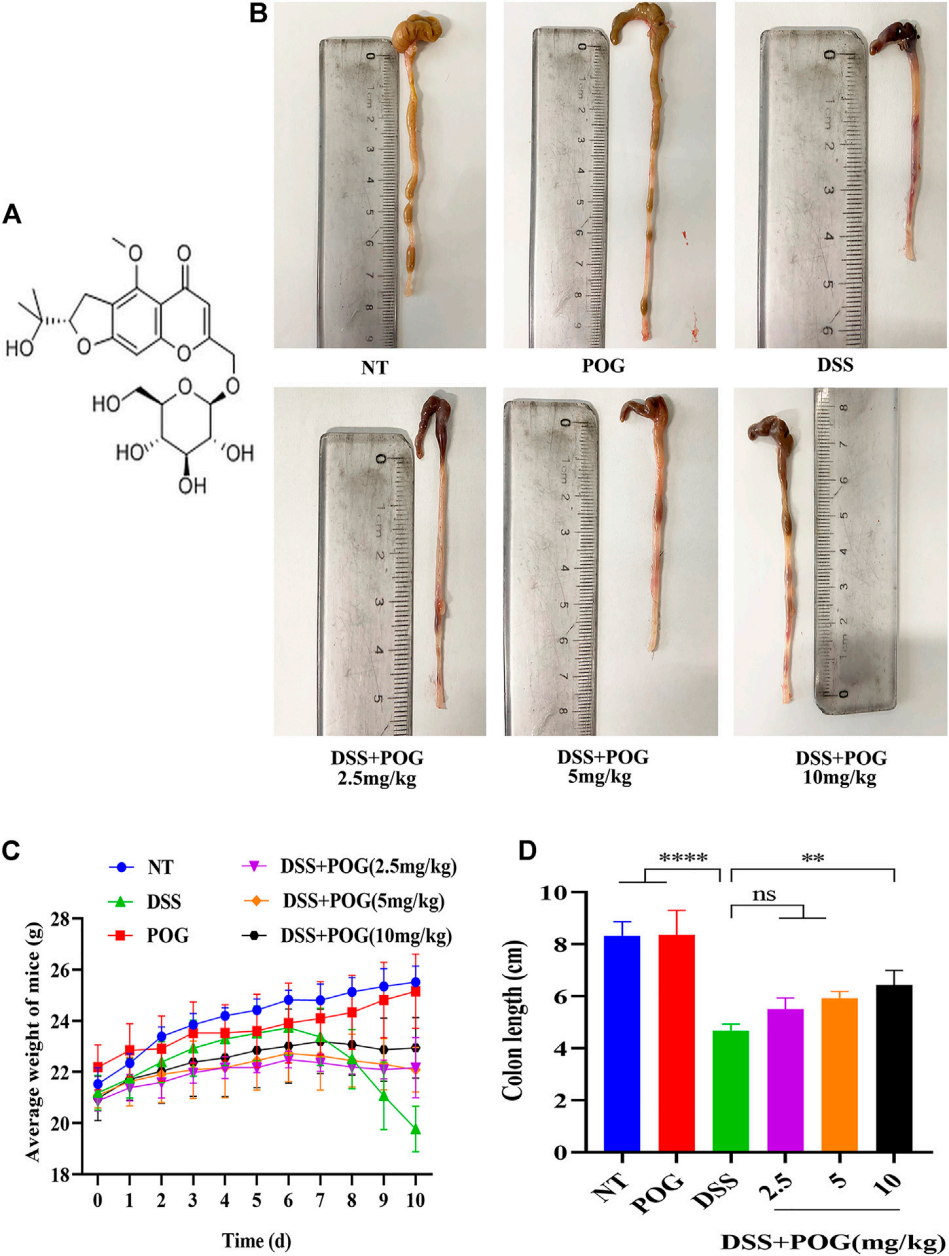
Protective effect of POG on DSS on colonic injury in mice. **(A)** POG structural schematic. **(B)** A representative diagram of the length of the colon samples collected from each group. **(C)** Changes in the daily average body weight of each experimental group (*n* = 6). **(D)** Average length of the colon in each group.

To begin testing the protective efficacy of POG treatment in DSS-induced mice model of UC (Figure 1A). The images of the collected samples from the colon showed that the colon of the DSS group mice had hematochezia, along with significantly shortened colonic length (Figure 1B). The weight of the mice was recorded daily, colon samples were collected, and the length was measured (Figure 1C). DSS mice had a shorter average colon length than other groups, and the DSS + POG group (10 mg/kg) significantly improved the damage of DSS to the colon length of mice, as seen in Figure 1D.

### The Specific Protective Properties of POG on the UC Mice Model

**FIGURE 2:**
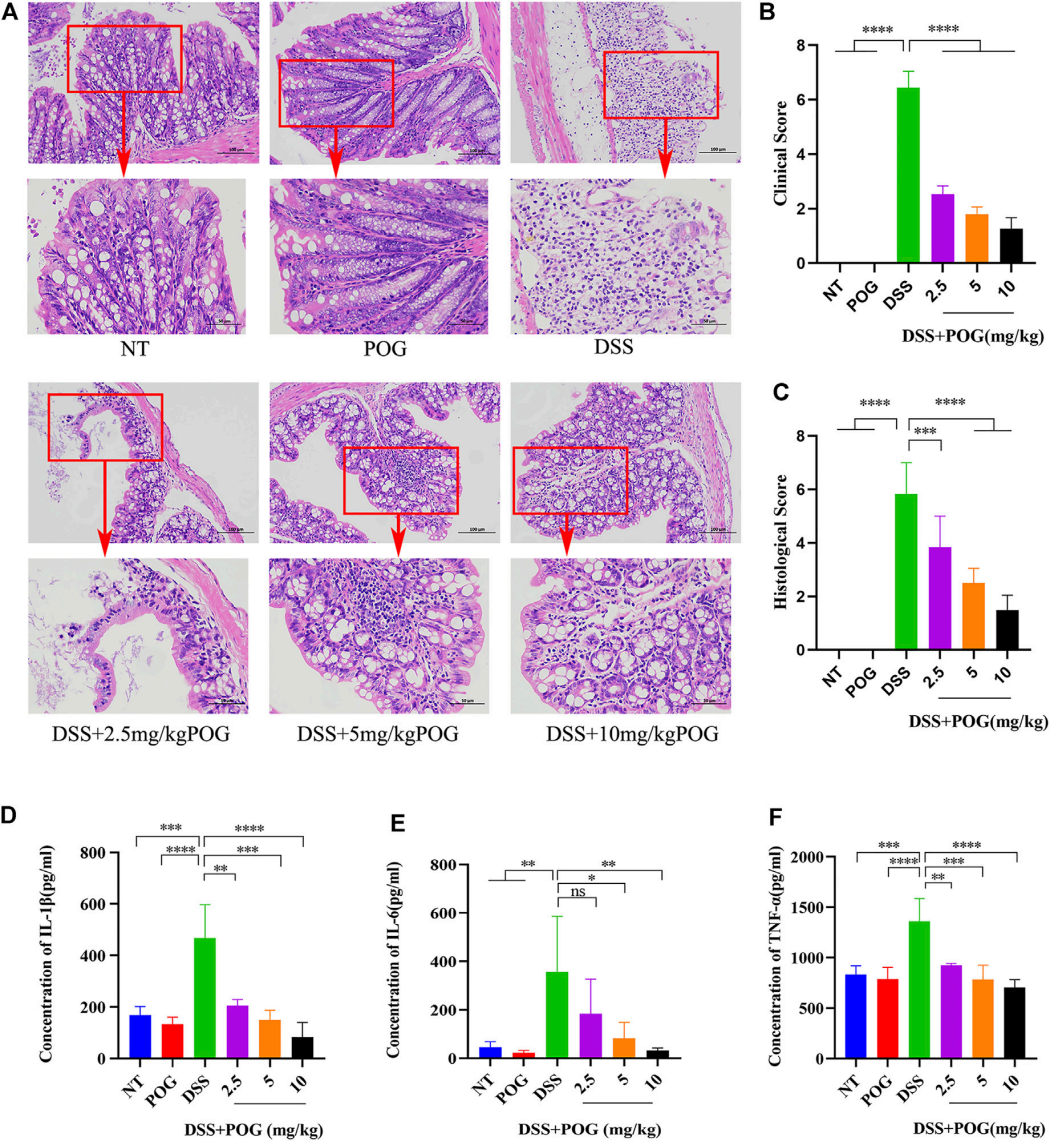
POG protects the intestinal tissue from damage and inhibits the production of related inflammatory factors. **(A)** Colonic tissue H&E staining images of all groups. **(B)** DSS-induced clinical scores of ulcerative colitis in mice. **(C)** Colon histological score. **(D–F)** Histogram of inflammatory cytokine expression (IL-1β, TNF-α, IL-6). Data are means ± SD. *p < 0.05, **p < 0.01, ***p < 0.001, ****p < 0.0001.

In order to explore the protective effects of POG on the UC mice model, mice colon tissue samples were stained with hematoxylin-eosin. As a result, DSS mice showed fewer colonic goblet cells, lost colonic crypts, significantly damaged intestinal mucosa, and exhibited typical inflammatory tissue edema infiltration relative to other groups (Figure 2A). Histological and clinical scores showed that the DSS + POG group (2.5, 5, and 10 mg/kg) significantly decreased the histological and clinical scores of DSS mice dose-dependently (Figures 2B,C). In addition, levels of inflammatory factors (TNF-ɑ, IL-6, and IL-1β) in each group of mice were inhibited by POG within DSS mice; this concluded effective control of the development of inflammation by POG (Figures 2D–F).

### POG Treatment Blocks the Inflammatory MAPK, AKT, and NF-κB Signaling Pathways in Mice With Ulcerative Colitis

**FIGURE 3:**
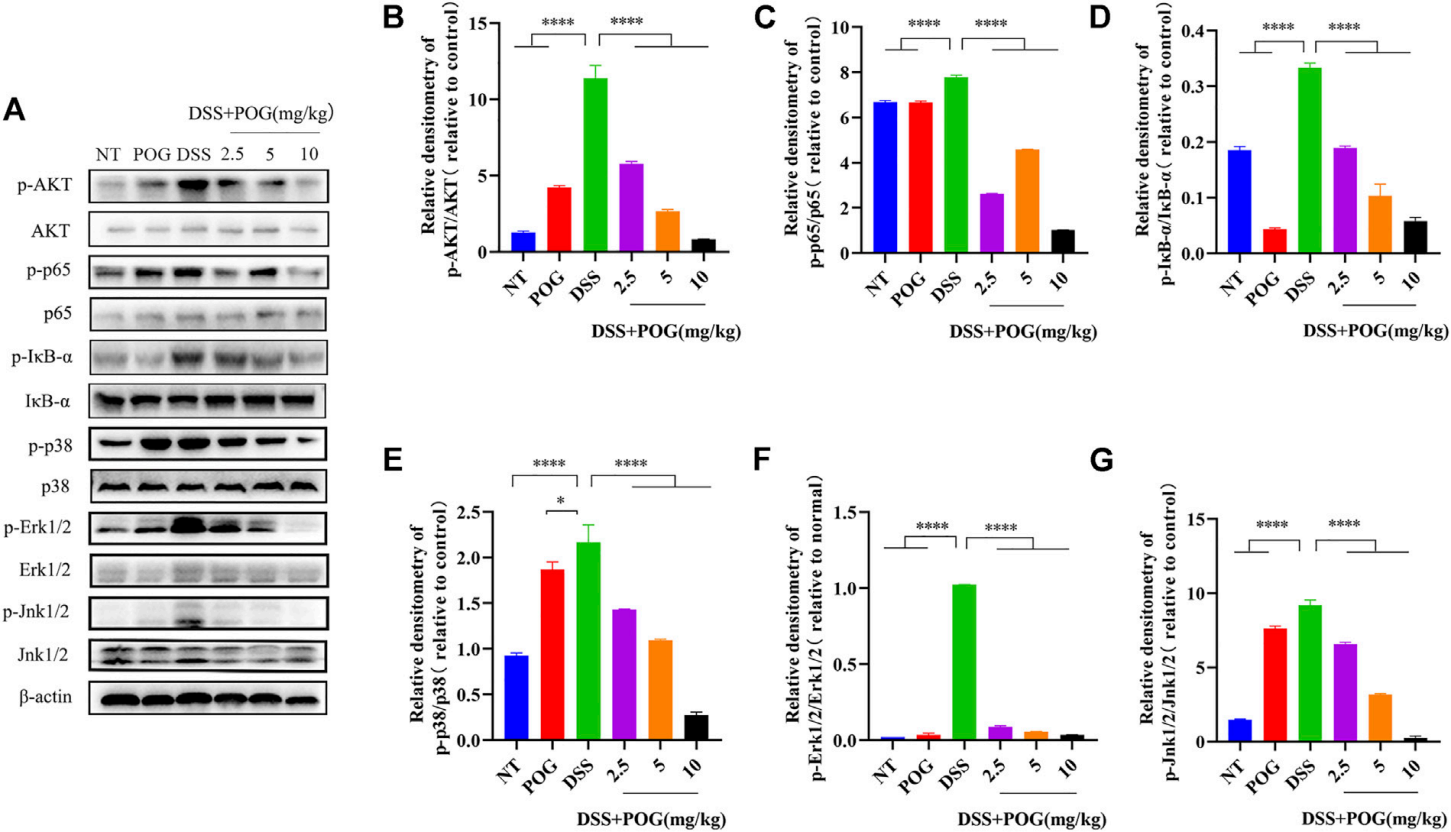
**(A)**POG effectively controlled the expression of protein bands phosphorylated by AKT, NF-κB, and MAPK signaling pathways in UC mice. **(B–D)** AKT, IκB-α, and p65 protein phosphorylation of all groups were measured through WB assay. **(E–G)** The phosphorylation of P38, ERK1/2, and JNK1/2 in the MAPK pathway was evaluated by Western Blot. Data are means ± SD (*n* = 3). *p < 0.05, **p < 0.01, ***p < 0.001, ****p < 0.0001.

In order to explore the protective mechanism of POG in UC mice, the mapped protein bands of MAPK, NF-κB, and AKT pathways were analyzed (Figure 3A). POG effectively hindered the phosphorylation activation of AKT, p65, and IκB-α proteins in NF-κB and AKT pathways (Figures 3B–D). Moreover, P38, JNK1/2, and ERK1/2 had significantly increased phosphorylation within the MAPK pathway in the DSS group, while POG administration reversed the above effect (Figures 3E–G). Therefore, POG suppressed MAPK, NF-κB, and AKT pathway activation, while alleviating inflammatory injury of mice colon.

### The Effect of POG on Colon Immune Barrier Protein Expression in Mice

**FIGURE 4:**
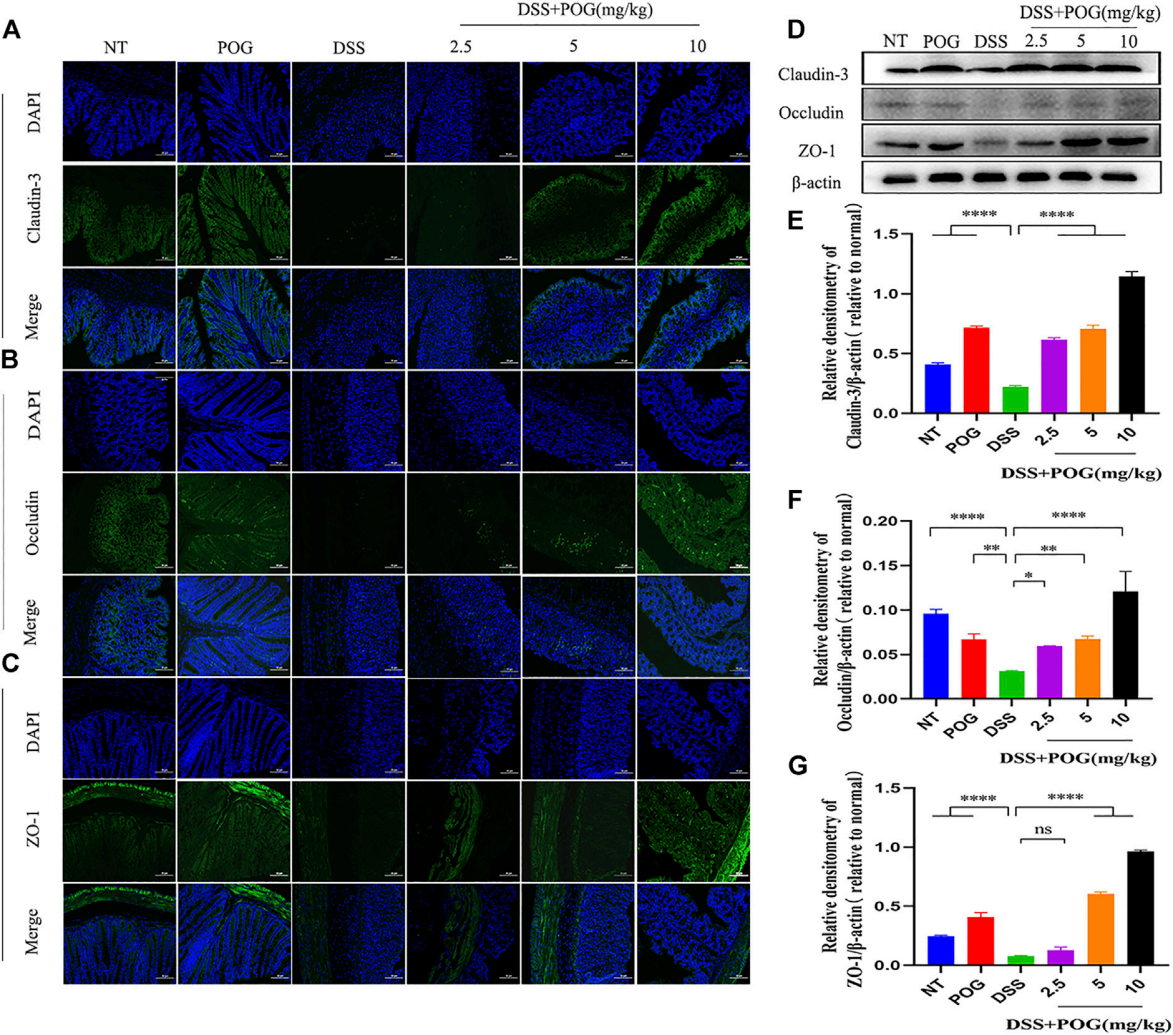
The effect of POG on tight junction protein induced by DSS in mice UC model. **(A–C)** The three TJ proteins Occludin, ZO-1, and Claudin-3 were analyzed through IF staining (20×); scale bar: 50 µm. **(D–G)** Western Blot method was used to detect the three protein levels of Occludin, Claudin-3, and ZO-1. Data are means ± SD (*n* = 3). *p < 0.05, **p < 0.01, ***p < 0.001, ****p < 0.0001.

The tight connectivity of intestinal tight junction (TJ) proteins is an important factor in forming the intestinal immune barrier. Immunofluorescence staining of the three tight junction proteins, namely, Occludin, Claudin-3, and ZO-1, and Western Blot experiments showed reduced expression levels in each group of colon samples (Figures 4A–G). This fully proved that DSS mice had the lowest TJ protein levels compared to other groups. It demonstrated that POG effectively alleviated the decrease of Occludin, Claudin-3, and ZO-1 protein levels in the DSS-induced mice ulcerative colitis model.

### Effect of POG Treatment on the Intestinal Microbiome

**FIGURE 5:**
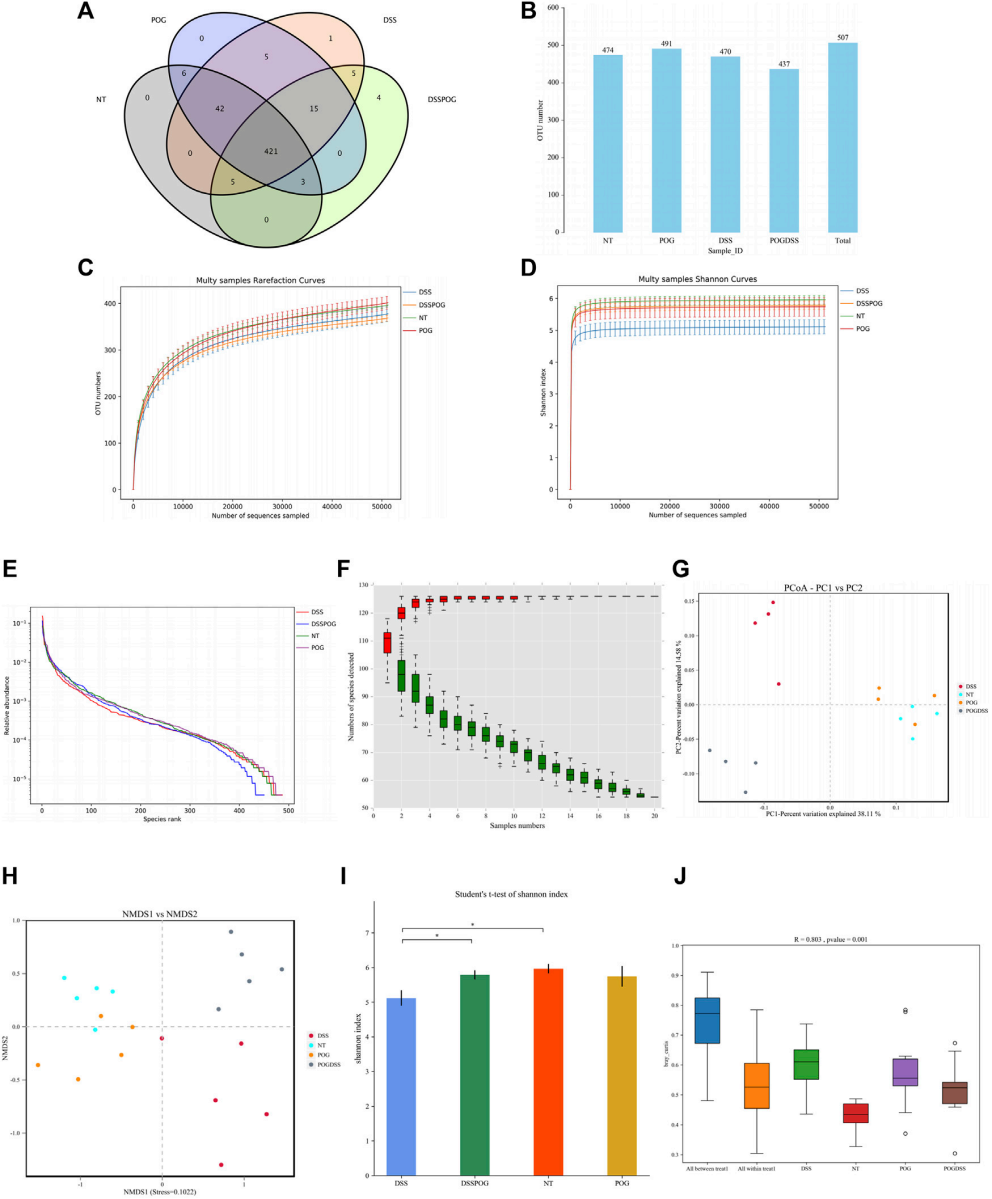
Role of POG in the intestinal flora. **(A)** Characteristic Venn diagram between groups. **(B)** Distribution of UTO numbers in each group **(C)** Dilution curve. **(D)** Shannon curves. **(E)** Rank and curve. **(F)** Species accumulation curves. **(G)** PCoA score chart. **(H)** NMSD score chart. **(I)** Alpha diversity index difference between groups histogram. **(J)** ANOSIM analysis box plot.

In this 16S rDNA, HTS was carried out to evaluate differences in gut microbiota. The differences in the Venn diagram and auto-number diagram were not significant among the four groups (Figures 5A,B). There were adequate sequencing data for reflecting species diversity in the sample (Figure 5C). Relative to DSS mice, mice in the rest three groups show more species of bacteria in the flora, and this richer species indicates that the test samples cover most of the microbial species information and sufficient sample size (Figures 5D–F). PCoA analysis and NMDS analysis show that the closer the distance on the coordinate map, the higher the similarity. Some studies believe that the effect of NMDS is better than PCA and PCoA. A stress value < 0.2 reflected the partial reliability of NMDS analysis (Figures 5G,H). Alpha (α)-diversity analysis revealed higher flora species diversity in three groups except for the DSS group (Figure 5I). Based on the analysis of similarities, that is, ANOSIM analysis, beta diversity did not present obvious differences among the four groups (Figure 5J).

**FIGURE 6:**
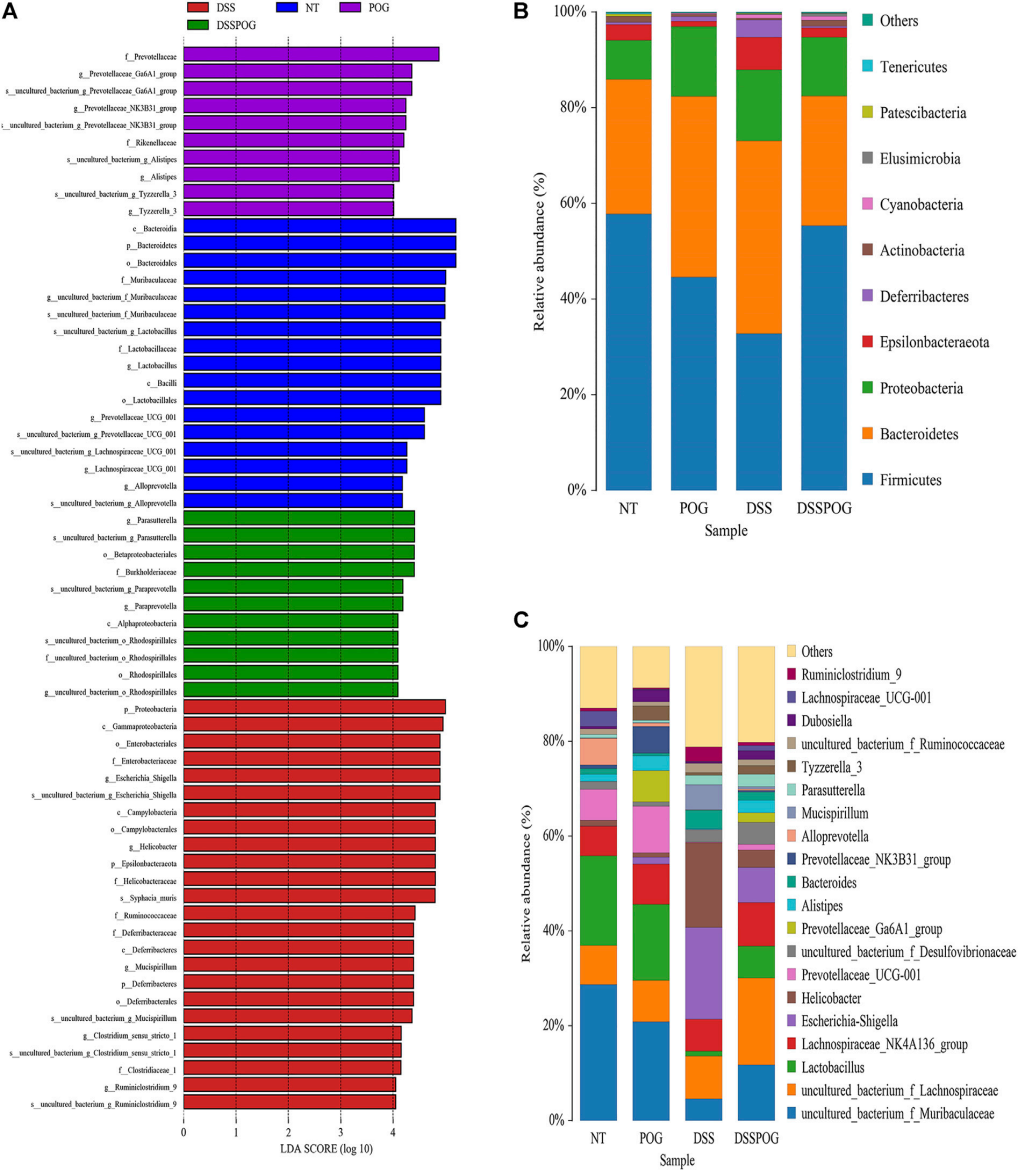
Intestinal microbiome composition analysis of mice in each group. **(A)** Histogram of LDA value distribution. **(B)** The top ten phyla of microbial composition in four groups of mice colon samples. **(C)** The first twenty genera of microorganisms in four groups of mice colon samples.

**FIGURE 7:**
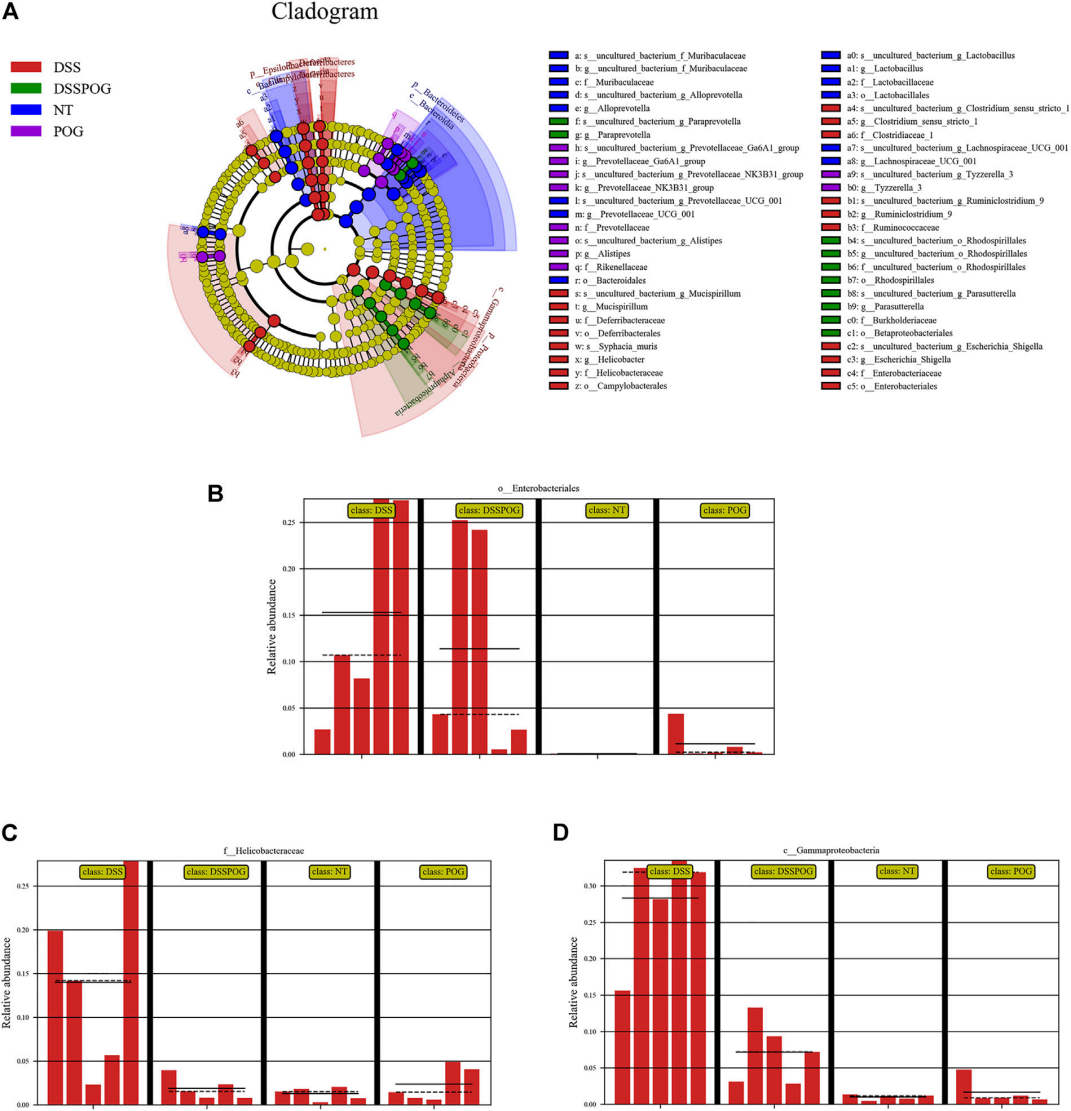
Abundance analysis of the intestinal microbial composition of mice in each group. **(A)** LEfSe analysis evolutionary branching diagram. **(B–D)** Relative Enterobacteriales, Helicobacteraceae, and Gammaproteobacteria abundance.

Histogram of LDA value distribution showed that intestinal microbial species were significantly different between the four groups (Figure 6A). By analyzing the relative microbial abundance of phyla and genus level, the DSS group had decreased species richness of *Lactobacillus*, Firmicute, and Bacteroidetes compared with the POG group, whereas the level of Proteobacteria markedly decreased in DSS (Figures 6B,C). The cladogram was plotted from linear discriminant analysis effect size (LEfSe) analysis indicating a significant difference in all four groups. Diverse colors in cladogram represent diverse groups, whereas distinct node colors reflect the degree of microbiota in the grouping. Therefore, microbial levels were significantly different across the four groups (Figure 7A). DSS group had a markedly increased abundance of Enterobacteriales, Gammaproteobacteria, and Helicobacteraceae compared with the remaining three groups, which indicated that DSS-mediated UC model mice had disrupted gut microbial population structure (Figures 7B–D). POG positively regulates the colonization of intestinal flora, protects beneficial flora, and maintains the homeostasis of the intestinal environment.

### POG on Cell Activity Detection and Inhibition of Related Inflammatory Factors

**FIGURE 8:**
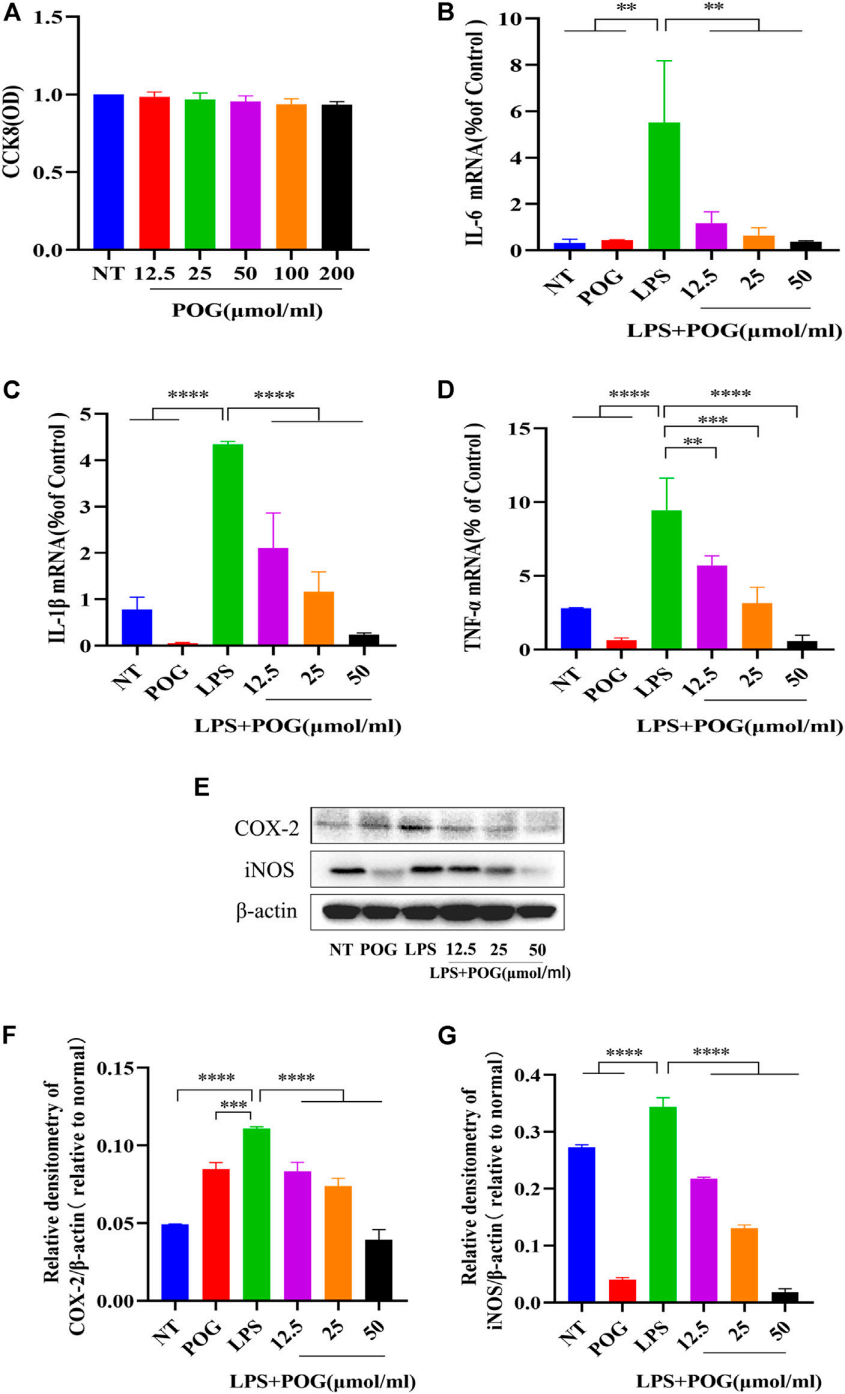
POG treatment suppresses inflammatory cytokine expression within LPS-treated RAW264.7 cells. **(A)** The activity of CCK8 cells was determined to affect RAW264.7 cells within the range of POG (12.5, 25, 50, 100,200 μmol/L). **(B–D)** qRT-PCR extraction of cellular mRNA for detecting IL-1β, TNF-α, and IL-6 expression. **(E–G)** iNOS and COX-2 protein levels measured through WB assay. Data were means ± SD (*n* = 3). *p < 0.05, **p < 0.01, ***p < 0.001, ****p < 0.0001.

The effect of POG on affected LPS-mediated RAW264.7 cells was examined *in vitro*. Firstly, the CCK8 method tests whether POG between 12.5 and 200 μmol/mL has toxic side effects on RAW264.7 cells (Figure 8A). RAW264.7 cells were subject to 1 h POG pretreatment, followed by 12 and 24 h LPS stimulation, respectively. Further mRNA and protein expression analyses were performed. qRT-PCR analysis indicated POG suppresses IL-1β, TNF-α, and IL-6 levels dose-dependently (Figures 8B–D). Meanwhile, protein bands showed that POG effectively inhibited iNOS and COX-2 upregulation within LPS-mediated RAW264.7 cells (Figures 8E–G).

### The Effect of POG on LPS-Induced MAPK, AKT, and NF-κBp65 Signaling Pathways in RAW264.7 Cells

**FIGURE 9:**
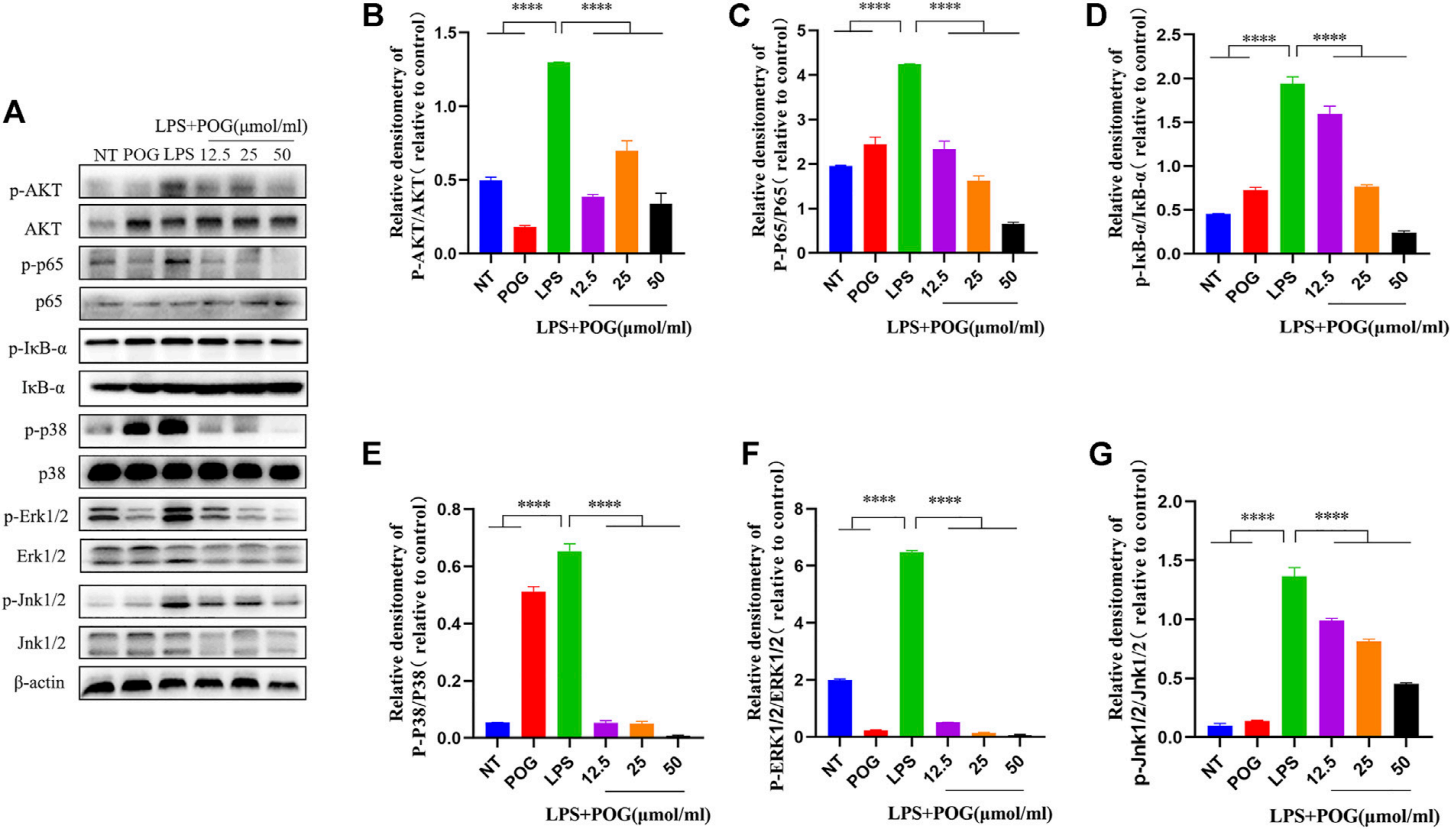
POG treatment inhibits MAPK, NF-κB, and AKT pathway activation within LPS-treated RAW264.7 cells. **(A)** Protein phosphorylation of MAPK-, AKT-, and NF-κB-related pathways was assessed by Western Blot. **(B–G)** Express MAPK, AKT, and NF-κB signal pathway phosphorylated protein levels and calculate the corresponding phosphorylated protein ratio. Data are means ± SD (*n* = 3). *p < 0.05, **p < 0.01, ***p < 0.001, ****p < 0.0001.

To further investigate the effect of POG treatment on inflammation-related pathways, we extracted the proteins within LPS-mediated RAW264.7 cells for analyzing protein phosphorylation levels in MAPK, NF-κB, and AKT pathways (Figure 9A). According to the results, POG treatment significantly inhibited phosphorylation of AKT, IκB-α, and p65 proteins within RAW264.7 cells (Figures 9B–D). Meanwhile, POG treatment also decreased P38, JNK1/2, and ERK1/2 phosphorylation within the MAPK pathway (Figures 9E–G).

## Discussion

Although UC is common, the initial symptoms are frequently overlooked (Ng et al., 2013). The most common causes of UC are closely linked to gut microbiota and the intestinal mucosal barrier (Cooper et al., 2000; Ramos and Papadakis, 2019; Schreiner et al., 2019). During the experiment, it was clear that POG treatment significantly suppressed DSS-mediated inflammation in UC model mice and regulated the intestinal flora to achieve equilibrium while protecting the intestinal immune barrier from damage. Therefore, it has been demonstrated that POG can effectively prevent and treat ulcerative colitis in mice. Inflammation is an indication of UC. The clinical symptoms, inflammatory factor production, and histopathological characteristics of UC patients are similar to those of UC model rats, and the results of POG treatment contribute to the drug foundation for the treatment of human UC diseases (Cooper et al., 2000; Marafini et al., 2019). Currently, UC treatment options are limited. The majority of them are conservative treatments involving drugs and biological agents. Surgical treatment should be considered in severe cases. Most patients are dissatisfied with traditional drug treatment schemes at the moment, so they must seek more effective, innovative, and efficient treatment to replace traditional therapy. This study discovered that POG protects the pathological damage and shortening of the colon of diseased mice throughout the pathogenesis of the DSS-mediated UC mice model and greatly improves mice weight maintenance. The findings indicate a dose-dependent POG effect in the treatment of UC model mice. The pathogenic mechanism of UC is still unknown. Many factors can cause UC. There is much evidence that immune cells and non-immune cells interact with each other when the intestinal environment’s homeostasis is out of balance, causing colon tissue damage and inflammation.

It is an effective treatment for inhibiting the production of inflammatory cytokines (TNF-α, IL-6/12/23) and the development of inflammation (Marafini et al., 2019). As a result, this study examined inflammatory cytokine levels in the UC mice model and discovered that POG could suppress TNF-α, IL-6, and IL-1β levels. Inflammatory factors interact with inflammation to speed up the progression of UC disease, and they are one of the key elements driving the inflammatory response (Bonaventura et al., 2015; Marafini et al., 2019; Dvornikova et al., 2021). This study looked at how POG affected LPS-mediated RAW264.7 cell inflammation and discovered that POG also significantly reduced IL-1β, TNF-α, and IL-6 levels in RAW264.7 cells. According to the above findings, POG can reduce the severity of UC disease by inhibiting the levels of the three inflammatory cytokines mentioned above.

The damage to the intestinal immune barrier is a key factor in the progression of UC, and the intestinal immune barrier is formed by the tight connection between cells. Goblet cells are mucus barrier epithelial cells that secrete trefoil peptides and mucins. Mucin MUC2 forms a layer of gel-like mucus in the intestinal epithelium, inhibiting bacterial colonization and translocation and thus maintaining intestinal homeostasis (Allaire et al., 2018; Hussain et al., 2019; Al Bander et al., 2020). Occludin, Claudin-3, and ZO-1 are tight junction proteins in the intestinal immune barrier that can maintain epithelial barrier integrity, while an increase in TJ protein permeability is associated with an increase in UC incidence. The study of these three TJ proteins revealed that POG protected the intestinal immune barrier, implying that Occludin, ZO-1, and Claudin-3 levels were lower in the DSS-induced UC mice model than in the other groups. This demonstrates that POG significantly regulates the aberrant TJ protein levels in the mice UC model and effectively reduces cell tissue permeability. It also heals the intestinal mucosa and alleviates UC symptoms. Prior studies have consistently reported that increasing Occludin and ZO-1 protein expression can reduce UC-induced intestinal mucosal damage (Nunes et al., 2019).

The intestinal flora is an important component of the intestinal epithelial barrier and is essential for maintaining internal environment homeostasis (Li et al., 2021). Intestinal flora imbalance can hasten the progression of UC (Al Bander et al., 2020). Furthermore, there are significant differences in the abundance and variability of the intestinal flora of UC patients *versus* healthy subjects (Liu Z. et al., 2020). In the course of UC research, it has been discovered that inhibiting or increasing inflammatory factors can alter the balance of the intestinal environment, thereby destroying or protecting the growth of the intestinal microbial flora (Wen and Duffy, 2017). As a result, we assessed the intestinal microbes of various groups, and POG had a significant effect on the levels of certain species. For example, in the DSS + POG group, the species richness of *Lactobacillus* and Firmicute increased significantly compared to DSS mice, while the species richness of Enterobacteriales, Helicobacteraceae, and Gammaproteobacteria decreased significantly. Relevant research has shown that the Firmicutes/Bacteroidetes (F/B) ratio is critical for maintaining intestinal homeostasis. If the F/B ratio rises, the majority of them appear in obese patients, and if the F/B ratio falls, the majority of them can be found in patients with enteritis (Stojanov et al., 2020). According to this research result, the DSS group has a lower F/B ratio than the POG + DSS group, indicating that the POG treatment effect of the DSS + POG group is obvious. *Lactobacillus* is widely regarded as a beneficial bacterium with a critical effect on intestinal microbial composition and the ability to promote beneficial changes in gut microbial composition. It produces antibiotic compounds and prevents potentially pathogenic bacteria from colonizing the intestine (Hemarajata and Versalovic, 2012; Wang et al., 2019). In this test, the DSS + POG group had significantly higher *Lactobacillus* levels than the DSS group, indicating that POG exposure increases beneficial intestinal flora levels, balancing the relative stability of intestinal microbial species and protecting intestinal health. Related studies have found a link between microorganisms (Helicobacteraceae, Gammaproteobacteria, and Enterobacteria) and colorectal cancer, obesity, and prediabetes. It was discovered that lowering the activity of these three microorganisms suppresses the TLR4/NF-κB pathway, which prevents the development of prediabetic obesity and colorectal cancer (Ramos et al., 2018; Hu et al., 2020). POG can improve the species composition of beneficial bacteria in intestinal microorganisms, protect the intestinal immune system, suppress pathogenic bacterial levels, and decrease the risk of intestinal tumors.

**FIGURE 10:**
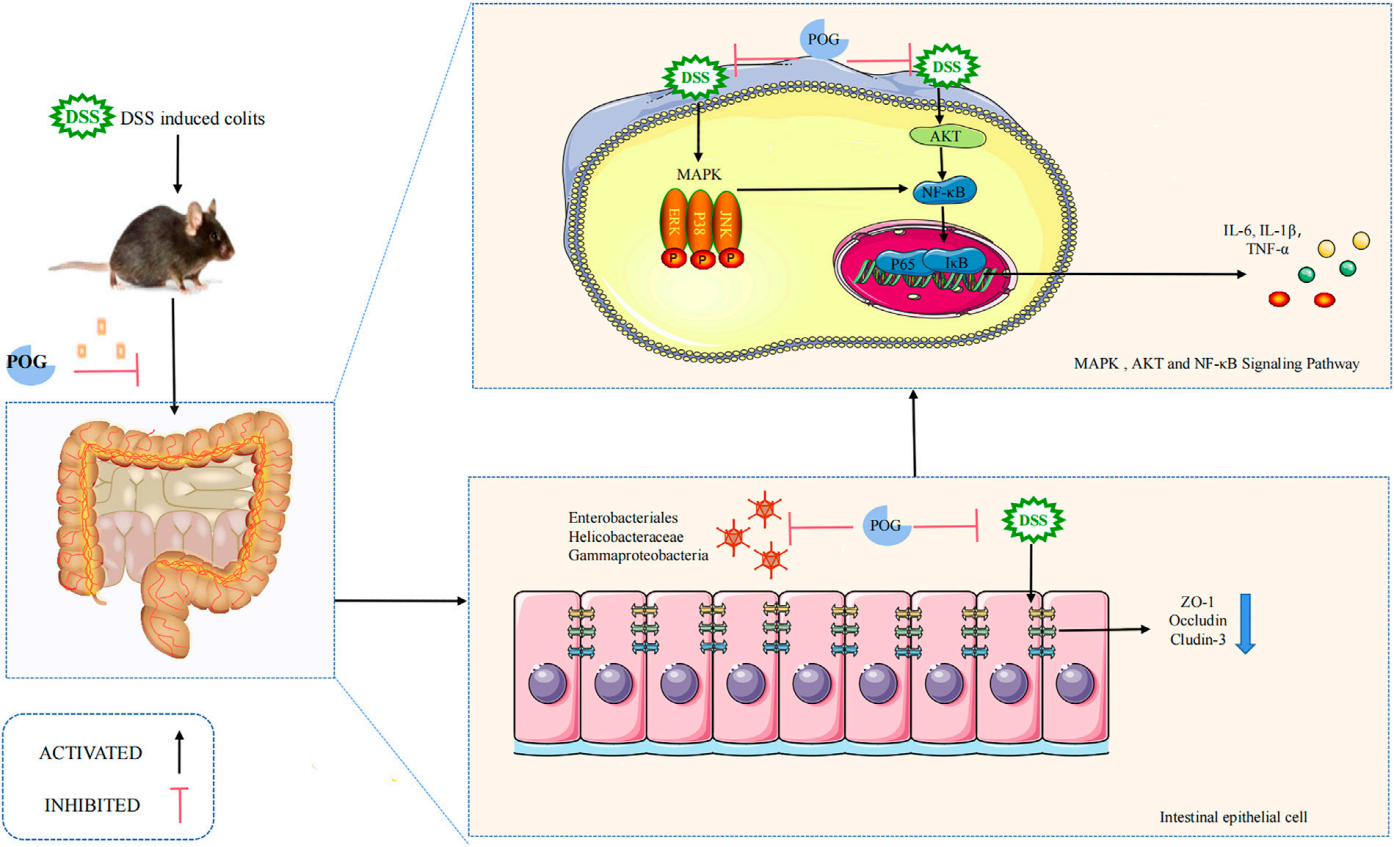
Mechanism of POG in inhibiting inflammation while maintaining epithelial barrier integrity within DSS-mediated UC. DSS successfully induced mice UC model. Colonic tissue damage was obvious as inflammatory factor levels elevated. POG can reduce DSS-induced UC and inhibit inflammatory factor production while alleviating colonic edema. POG modulates gut flora to maintain intestinal homeostasis while protecting the intestinal immune barrier from impaired avoidance but also inhibits AKT, MAPK, and NF-κB pathway activation, as well as inflammatory mediator expression (IL-1β, TNF-α, and IL-6).

The AKT pathway, which is located upstream of the NF-κB pathway, is involved in protein synthesis, proliferation, and survival throughout the process (Nennig and Schank, 2017; Yi et al., 2017). The majority of proteins in the NF-κB pathway can regulate inflammation, stress response, and the immune system. As a result, NF-κB serves as an activator for inflammatory factors involved in transcriptional communication (Guo et al., 2020; Guo et al., 2021). Furthermore, because the MAPK pathway is located upstream of the NF-κB pathway (Liu K. et al., 2020), its activation contributes to the activation of NF-KB as well as its nuclear transport, resulting in certain inflammatory reactions (Liu K. et al., 2020; Guo et al., 2021). Phosphorylation of AKT and the MAPK pathway facilitates NF-κB pathway activation (Nennig and Schank, 2017) while also mediating inflammatory cytokine levels (such as IL-1β, TNF-α, IL-6). As a result, we investigated how POG affected protein phosphorylation levels related to MAPK, NF-κB, and AKT pathways, including JNK1/2, ERK1/2, P38, P65, IκB-α, and AKT, using *in vitro* and *in vivo* experiments. POG has been found to reduce inflammatory cytokine production (such as IL-1β, TNF-α, and IL-6) in a dose-dependent manner by suppressing MAPK, NF-κB, and AKT pathway activation (Figure 10).

## Conclusion

In conclusion, POG has protective and alleviating effects on the DSS-induced UC model, and the underlying mechanism has been successfully explored. *In vitro* and *in vivo* experiments have demonstrated the efficacy and authenticity of POG treatment. POG inhibits inflammatory cytokine expression and related pathway activation, repairs the intestinal immune barrier, regulates the abundance of microorganisms in the intestinal environment, and has an excellent anti-inflammatory effect. POG is a drug that has the potential to be developed further in the future. POG is currently used in the research of colds, rheumatoid arthritis (RA), breast cancer, and other diseases in order to demonstrate the safety of POG and clarify its future clinical use. After comparing POG to traditional clinical drugs, we will follow up on the treatment of clinically willing patients and objectively evaluate clinical efficacy. We anticipate that POG will play an important role in UC disease.

## Data Availability

The original contributions presented in the study are included in the article/Supplementary Material, further inquiries can be directed to the corresponding author.
