# Supplementary material for: Protective Effect of Prim-O-Glucosylcimifugin on Ulcerative Colitis and Its Mechanism
Source: Front Pharmacol. 2022 May 18;13:882924. doi: 10.3389/fphar.2022.882924 (PMC9158503; doi:10.3389/fphar.2022.882924)

Figure 3

P-AKT AKT


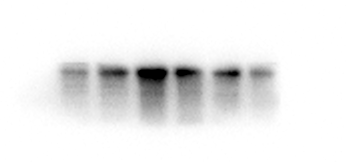

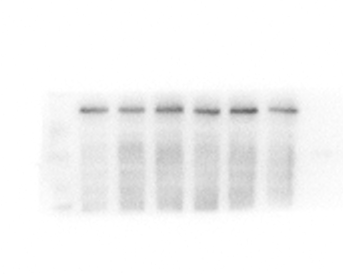


p-p65 p65


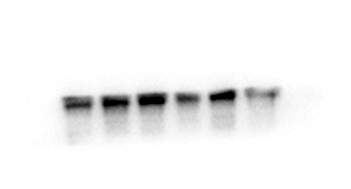

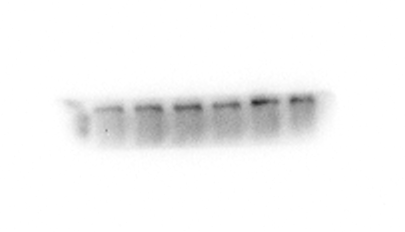


P-IκB-α IκB-α


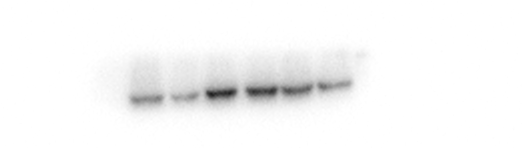

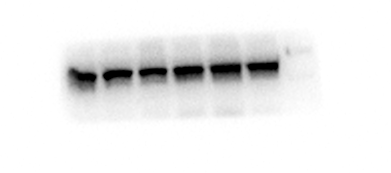


p-p38 P38


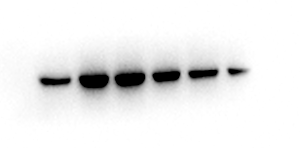

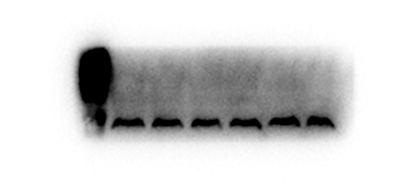


p-Erk1/2 Erk1/2


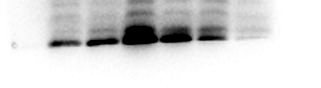

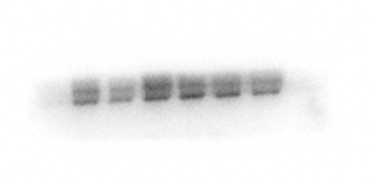


p-JNK JNK


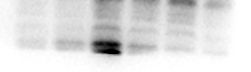

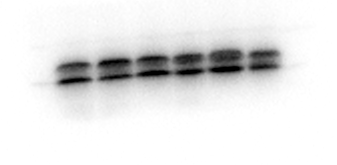


β-actin


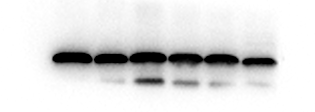


Figure 4

β-actin


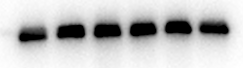


Claudin-3


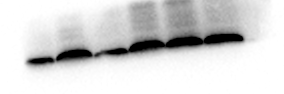


Occludin


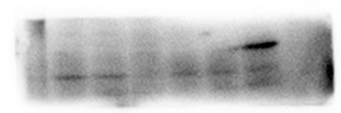


ZO-1


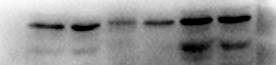


Figure 8

β-actin


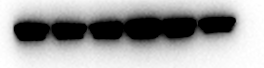


COX-2


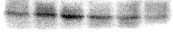


INOS


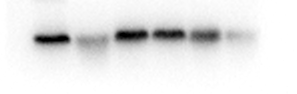


Figure 9

β-actin


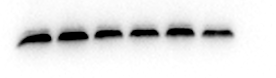


P-AKT AKT


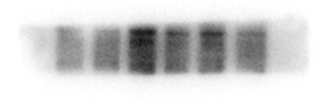

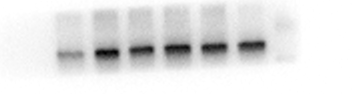


p-p65 p65


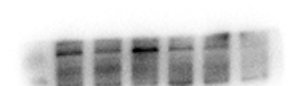

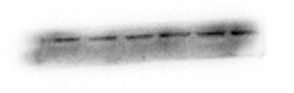


p-IκB-α IκB-α


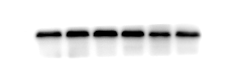

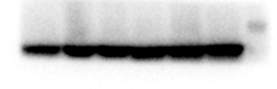


p-p38 P38


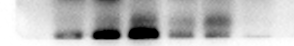

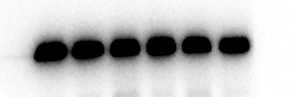


p-Erk1/2 Erk1/2


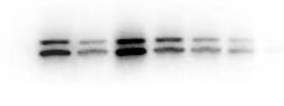

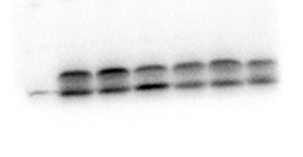


p-JNK JNK


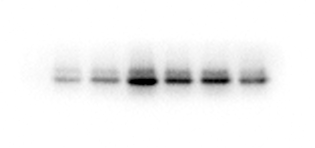

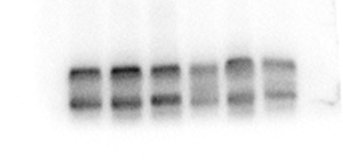

Supplement: Supplementary file 1 [file DataSheet1.doc]
